# A Scoping Review of Exosome Delivery Applications in Hair Loss

**DOI:** 10.7759/cureus.81152

**Published:** 2025-03-25

**Authors:** Sarah Schaffer, Lily Tehrani, Braeden Koechle, Prathmica Chandramohan, Brookie Hilburn, Kawaiola Cael Aoki, Robin J Jacobs

**Affiliations:** 1 Medicine, Dr. Kiran C. Patel College of Osteopathic Medicine, Nova Southeastern University, Fort Lauderdale, USA

**Keywords:** alopecia, exosome, extracellular vesicles, hair regeneration, laser therapy, microneedling

## Abstract

The objective of this scoping review was to understand the extent and type of evidence found in the current literature on the delivery mechanisms of exosome therapeutics and how these methods can work synergistically with existing treatments for alopecia. Alopecia is primarily characterized as non-scarring or scarring (cicatricial). In cicatricial alopecia, the hair follicles are irreversibly destroyed, causing permanent hair loss. In non-cicatricial alopecia, the hair follicles are undamaged, allowing for possible hair regeneration. Non-scarring alopecia includes androgenetic alopecia, telogen effluvium, and alopecia areata. Current treatments for non-scarring alopecia include oral minoxidil and spironolactone. Exosome therapeutics are a possible alternative treatment for non-scarring alopecia because of their regenerative properties in hair follicle stimulation, customizable size selection, and the potential to activate and down-regulate specific pathways that enhance hair growth. This review evaluates types and sources of exosome delivery as regenerative treatments for alopecia. A search of literature published in English from 2018 to 2023 was performed using the electronic databases EMBASE, Ovid MEDLINE, and Web of Science. Data from selected studies included specific details about the participants, concept, context, study methods, and key findings relevant to the review questions. Upon completion of the database search that yielded 1,087 citations, after removing 284 duplicates, 803 articles remained for assessment of eligibility. Finally, 16 studies were retained for inclusion. These studies explored one or more exosome delivery techniques, such as intradermal needle injection, microneedle patches, topical application, and topical application with a secondary assistive device. The therapeutic focus of these studies ranged from hair follicle regeneration and wound healing to spinal cord injury repair and collagen regeneration for cosmetic purposes. Most of the studies (14 out of 16) used exosomes derived from mesenchymal stem cells (MSCs), while others isolated exosomes from human adipose stem cells, macrophage cell lines, and dermal fibroblast cells. Of the 16 studies, all but two administered exosomes via microneedle patches. The findings suggest that intradermal microneedle patches are a promising method for delivering exosomes into tissues, particularly for the treatment of non-cicatricial alopecia. Exosome therapy shows strong potential for promoting hair follicle regeneration, supported by its proven efficacy in wound healing, spinal cord injury repair, and cosmetic applications. Among the various delivery methods explored, microneedle patches loaded with exosomes from MSCs emerged as the most effective for targeted delivery into tissues. These findings support exosome-based therapies for non-cicatricial alopecia.

## Introduction and background

Alopecia, or hair loss, can affect any sex at any age and poses a significant psychological burden. Roughly 40% of women reported that their hair loss contributed to marital problems, while 63% reported career problems [1]. Alopecia is categorized into non-cicatricial (nonscarring) and cicatricial (scarring). Cicatricial alopecia is irreversible and has no potential to respond to regenerative treatments. The two most common forms of non-cicatricial alopecia are androgenetic alopecia and alopecia areata [2]. Androgenetic alopecia is the most common form of progressive hair loss in the United States, affecting 80% of Caucasian men and 40% of Caucasian women by age 70. Its pathogenesis involves hypersensitization to the androgen, dihydrotestosterone (DHT), that binds to receptors on hair follicles. The unrestrained stimulation of these receptors leads to miniaturization, or reduced follicle size and thinning of hair [3,4]. Alopecia areata affects 2% of the world population; it is an uncontrolled autoimmune response resulting in T-cell-mediated cytokine inflammation, which destroys hair follicles and prevents hair growth [5]. In the case of alopecia, a range of treatments are available, but they are limited in their effectiveness and tolerability. This review thus explored the potential of exosome therapy as a novel treatment method for non-cicatricial forms of alopecia, focusing on androgenetic alopecia and alopecia areata. Also discussed are the mechanisms of action, current treatment options, challenges, and the need for further research in utilizing exosomes to enhance hair follicle regeneration and improve alopecia treatments. 

Current treatments 

Current topical treatments for androgenetic alopecia include minoxidil, which increases blood flow to the follicles by inhibiting potassium-ATPase channels, and finasteride, an oral 5-alpha-reductase inhibitor that obstructs DHT activity, leading to hair regrowth. Minoxidil and dutasteride (which has a similar mechanism to finasteride) can also be given orally to patients. Other oral medications, including cyproterone and spironolactone, exert anti-androgenic effects. Cyproterone acetate is a gonadotropin and 5-alpha-reductase inhibitor. Spironolactone reduces the amount of testosterone secreted by the adrenal glands by inhibiting 17a-hydroxylase and desmolase [6]. Non-pharmaceutical low-level laser therapy uses low-fluence red laser light to stimulate the scalp. This process triggers the release of nitric oxide, which helps vasodilate blood vessels, thereby increasing blood flow to the hair follicles. Microneedling involves repetitive skin puncturing with needles, which stimulates dermal papillary cells' wound-healing mechanism; it is often used with platelet-rich plasma (PRP) or topical minoxidil. PRP contains growth factors that can contribute to increased hair growth [6]. For alopecia areata, corticosteroids reduce autoimmune attacks against hair follicles and can be applied topically or intralesionally for mild forms [7]. Oral JAK inhibitors are another treatment option; they work by targeting T cells, which help hair follicles transition back into the anagen (growth) phase [6]. 

To treat alopecia, the method of drug delivery is of significant consideration. Oral medications are systematically dispersed and may lead to unwanted side effects. For example, oral finasteride can cause erectile dysfunction in men and is cautioned in its use for women as it may cause infertility [6]. Corticosteroid injection therapy is another treatment modality with considerable side effects, which vary based on the treatment duration. Short-term corticosteroid use is associated with cutaneous effects, hyperglycemia, and neuropsychological effects, while long-term use has been associated with more severe side effects such as adrenal insufficiency [8]. Cutaneous atrophy has also been found as a potential side effect [9]. 

Excessive sebaceous content can entrap hair follicles and prevent topical applications from penetrating them, rendering them less effective [10]. Therefore, procedures like microneedling, which can penetrate the stratum corneum, are more effective when combined with other topical treatments for alopecia than when used alone [10]. Effective treatments for alopecia require further research.

Exosomes 

Exosomes were first observed in 1946 and are vesicles secreted by cells that carry cellular information that can influence other cells' growth, development, and functions [11,12]. Communication between exosomes and cells occurs through several signaling pathways and cell receptors such as toll-like receptor-2 (TLR-2), toll-like receptor-4 (TLR-4), lectin-like oxidized low-density lipoprotein receptor (LOX-1), the mitogen-activated protein kinase pathway (MAPK), and the natural killer group 2D signaling pathway (NKG2D) [12]. Exosomes can be collected for therapeutic use from multiple sources, such as aortic endothelial cells and mesenchymal-derived exosomes from the umbilical cord, bone marrow, and adipose tissue [13]. Because of the vast availability of mesenchymal-derived exosomes, they have been the focus of exosome research for alopecia treatment and other conditions like atopic dermatitis, melanoma, wound healing, brain injury, cardiac injury, hepatic diseases, and bone regeneration [14,15]. Exosomes are 40 to 160 nm in diameter and are small enough to seep into the hair follicle [10,16].

Exosomes can be isolated and modified to deliver proteins, DNA, RNA, or drugs, and the surface receptors can be tailored to direct exosomes to desired tissues and cells [13]. Therefore, exosomes can be loaded with tailored contents and delivered to target cells to influence gene expression and cell differentiation. This targeted form of therapy allows for minimal side effects with a simultaneous increased therapeutic concentration dose [17]. Exosomes have been discovered to increase hair dermal papilla cells' expression of angiogenic growth factors, like vascular endothelial growth factor (VEGF), and growth hormones, like insulin-like growth factor-1 (IGF-1), leading to hair growth [18]. Exosome therapeutics are a possible alternative treatment for non-scarring alopecia because of their regenerative properties in hair follicle stimulation, customizable size selection, and the potential to activate and down-regulate specific pathways that enhance hair growth [19].

Exosomes derived from dermal papilla cells (DPC-Exos) injected into the cutaneous hair follicle stimulate the proliferation of hair follicles in mice. DPC-Exos activates the expression of Sonic Hedgehog and B-catenin in outer root sheath cells while also enhancing the proliferation of ORSCs, thereby simultaneously accelerating the onset of the anagen phase while delaying the catagen (transition) phase of hair follicles [20]. Exosomes derived from adipose-derived stem cells (ADSC-Exos) have been found to down-regulate miR-22, a post-transcriptional regulator that promotes the transition of anagen to catagen in the hair cycle, causing hair follicle regression and TNF-α in DPCs while activating the Wnt/B-Catenin signaling pathway [21]. 

Exosomes can be tailored to deliver specific contents like proteins, DNA, RNA, or drugs to target cells, offering a potential method to enhance the efficacy of current alopecia treatments. By utilizing exosomes, researchers aim to minimize systemic side effects and maximize the potency of topical and intradermal treatments, revolutionizing how alopecia is managed.

Challenges with current therapies

While the potential use of exosomes is increasing, it comes with challenges. Exosomes can express protein and RNA profiles; however, different cells in the body can produce similar sets of proteins and miRNAs, making them difficult to distinguish unless there are cell-specific proteins. The lack of standardization poses a potential challenge in exosome therapy as the population of exosomes expressed from single cells is highly diverse and heterogeneous, causing a variation in the content concentrations. Determining the tissue of origin of an exosome is also difficult as their circulation throughout the body comes from various cell types with similar cargoes [22]. Additionally, the isolation and purification of exosomes lack standardization. Generally, multi-step centrifugation is a required step in the process of isolation. However, the exosomes isolated are often contaminated by non-exosomal extracellular vesicles (EVs) that are present in the body [22]. 

The exploration of exosome therapeutics for non-cicatricial alopecia, including alopecia areata and androgenetic alopecia, has revolutionized the field of dermatology as a promising avenue for hair follicle regeneration and adjunctive treatment to current techniques. The use of exosomes as a method to treat hair loss is a relatively new form of medicine that has only been explored in the past decade. However, exosomes as a potential alopecia therapy lack publicly available trials that analyze their safety and efficacy. There are no currently approved exosome products for the treatment of alopecia by the United States Food and Drug Administration, as several side effects still need to be examined [23]. The previously discussed treatments, delivery methods, and emerging findings about exosomes' ability to target tissues highlight the importance of exploring these themes to enable researchers to develop improved modalities in the treatment of alopecia. This scoping review aimed to report on the current literature on the delivery mechanisms of exosome therapeutics and how these methods can work synergistically with existing treatments for alopecia. 

## Review

Methods

This scoping review was conducted using the JBI methodology for scoping reviews [24].

Eligibility Criteria

This scoping review considered primary experimental human and animal studies (including randomized control trials and nonrandomized controlled trials) and descriptive observational studies (including case series, individual case reports, and descriptive cross-sectional studies). Both human and animal studies published between 2018 and 2023 were included as exosomes investigation in therapeutic use had only been investigated in recent years. The search was guided by the review questions “What evidence exists on the delivery mechanisms of exosome therapeutics,” and “How can these methods synergize with existing treatments for alopecia, with a focus on the types and sources of exosome delivery as regenerative therapies?”

Search Strategy

An initial limited search of EMBASE, Ovid MEDLINE, and Web of Science was conducted. This was undertaken to identify articles on the topic. The text words contained in the titles and abstracts of relevant articles and the index terms used to describe the articles were used to develop a full search strategy for EMBASE, Ovid MEDLINE, and Web of Science. The search strategy, including all identified keywords and index terms, was adapted for each included database and/or information source (see Table 2 in the Appendix). The reference list of all included sources of evidence was screened for additional studies. Studies published in the English language were included due to researcher language limitations. 

The database search yielded 1,087 citations. After removing 284 duplicates, 803 articles remained for eligibility assessment. Of those, 778 were excluded for the following reasons: no mention of exosome delivery (n=244); not primary research (n=281); topic not relevant (n=119); no abstract (n=75); wrong population (n=31); wrong study design (n=8); wrong outcome (n=9); out of date range (n=8); and duplicate studies not caught in first screening phase (n=3), leaving 25 articles for further assessment. Of those 25, 8 were excluded for only having an abstract or the full text not being available, leaving 17 articles for further critical appraisal. From the appraisal, one article was excluded due to an unacceptable level of bias, leaving 16 articles eligible for the final review.

Data Charting and Extraction Process 

Rayyan, a web-based software tool designed to assist with the scoping review screening and selection processes, and Excel (Microsoft Corporation, Redmond, WA) for data extraction charting were used for data management throughout this scoping review. Through an iterative process, team members independently charted the data, discussed the results, and continuously updated the data-charting form. The information extracted focused on the article’s purpose, study population, sample, methods, limitations, and key findings of the final articles included in the review. 

Specifically, data were extracted by two independent reviewers using Rayyan. The extracted data included details about the participants, concept, context, study methods, and key findings relevant to the review question. The data results were obtained from articles and then sorted and organized using Excel. The information of interest encompassed the study's purpose, the source and type of exosomes, administration methods, the tissue medium they were introduced into, and the population for in vivo experiments. The data extracted from each included evidence source was adjusted and refined as needed. Any disagreements on whether to include or exclude an article were discussed among two reviewers; there was no need for an additional reviewer. The final draft underwent editing and review by the remaining authors. Any global discrepancies were addressed collectively among all authors through discussion and debate.

Source of Evidence Selection

Following the search, all identified citations were collated and uploaded into EndNote Version 21 (Clarivate, Philadelphia, USA), and duplicates were removed. Following a pilot test, titles and abstracts were then screened by independent reviewers for assessment against the inclusion criteria for the review. Potentially relevant sources were retrieved in full, and their citation details were imported into the JBI System for the Unified Management, Assessment, and Review of Information (JBI SUMARI) [25]. The full text of each selected citation was assessed against the inclusion criteria by two independent reviewers. Reasons for excluding full-text sources of evidence for not meeting the inclusion criteria were recorded. Any disagreements that arose between the reviewers at each stage of the selection process were resolved with an additional reviewer.

Critical Appraisal of Individual Sources of Evidence

A thorough evaluation of the 17 articles was conducted using the critical appraisal tools developed by JBI. The appropriate checklist was applied to each article to assess research biases, overall coherence, and key factors influencing article quality. Two team members independently carried out a detailed, blind appraisal of the selected articles using the relevant JBI tools. The articles were then categorized based on their risk of bias: high, moderate, or low, according to their scores (below 50%, between 50% and 70%, and above 70%, respectively). Articles scoring above 70% were included, while those below 70% were excluded due to their higher risk of bias. Following this, the team engaged in a deliberative process to compare their appraisal scores. The relevance and quality of each article were carefully discussed, leading to a consensus on the final selection. One article was dropped as it did not meet the 70% threshold. As a result, all 16 articles were included in the final review.

**Figure 1:**
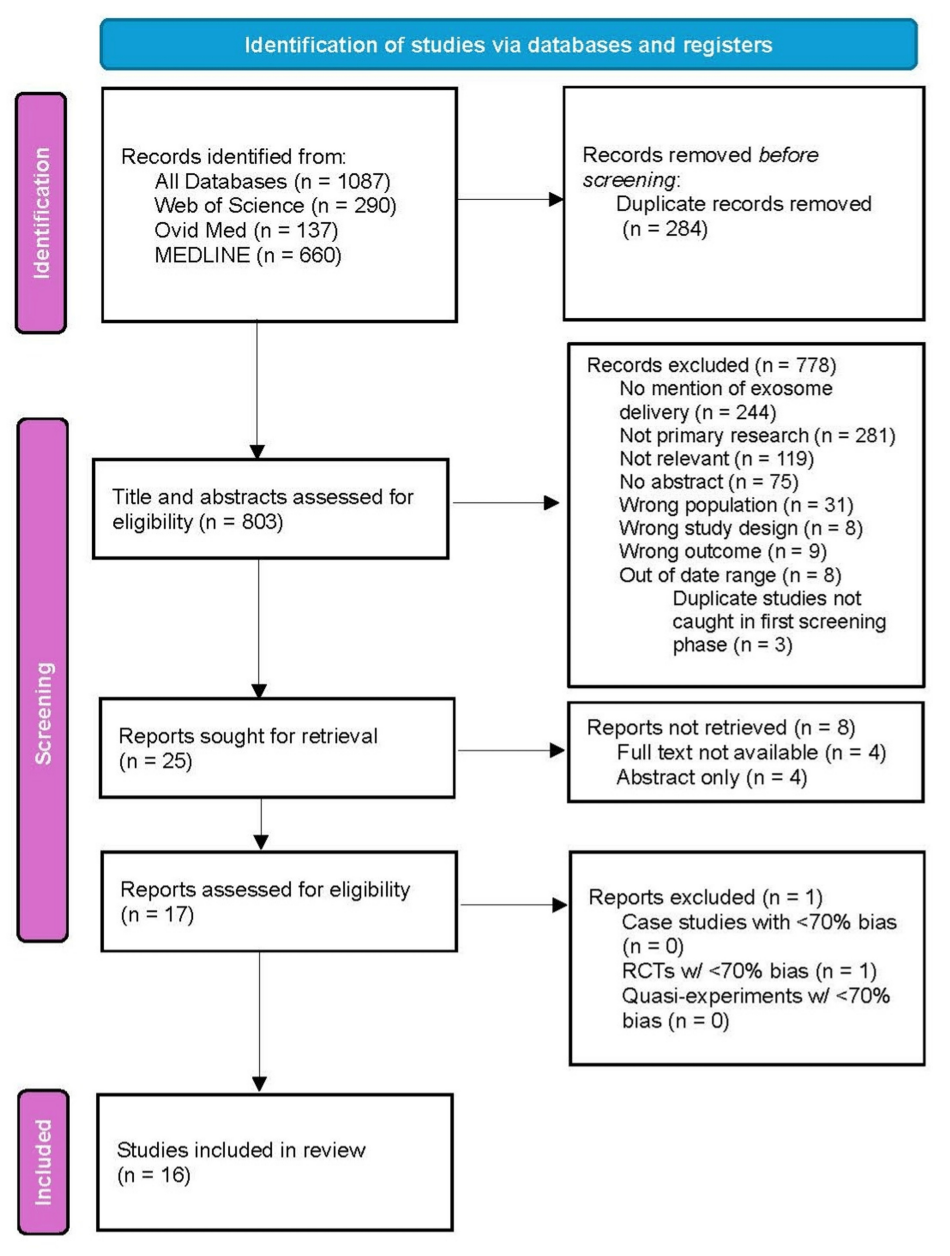
PRISMA flow diagram PRISMA: Preferred Reporting Items for Systematic Reviews and Meta-Analyses.

The selection results were reported and are presented in Figure 1 using the Preferred Reporting Items for Systematic Reviews and Meta-analyses extension for scoping review (PRISMA-ScR) flow diagram [26]. 

Results

Characteristics of the Studies

The review included studies that were published between the years 2018 and 2023. Each of the 16 articles employed one or more identified exosome delivery methods: microneedle patches/assays, intradermal injection by needle, topical application, and topical application assisted with a secondary device. Fourteen articles reported results from in vivo animal models (mice or rats); the other two articles conducted experiments with human subjects.

Exosome Sources

The exosomes were sourced from several origins. Seven studies used exosomes isolated from mesenchymal stem cells (MSCs), and three studies used exosomes derived from human adipose-derived stem cells [27-36]. Other exosome derivations used less frequently were from macrophages [37,38] and human dermal fibroblasts [39]. Three studies did not specifically use exosomes but did use isolated nanoparticles that possess a similar kinetic nature to that of exosomes [20,40,41]. For example, Lin et al. studied hair regrowth in rats by observing the delivery of nanoparticles with microneedle patches. These nanoparticles shared a similar size to that of exosomes, 100 nm- 150 nm [40]. The first nanoparticle type was composed of a poly lactic-glycolic acid copolymer with encapsulation of rapamycin, and the second nanoparticle type was made by cross-linking keratin with epigallocatechin gallate [40]. In the study by Zhao et al., miR-218 was loaded into lipid-polymer hybrid nanoparticles (LPNs), which were 141+14 nm in size, recall that exosomes are similar in size and can transport mRNA [37,42]. These hybrid nanoparticles were utilized to investigate the transdermal delivery of miR-218 into the scalp dermis of mice to promote dermal papilla cell proliferation and hair regrowth [42]. In a different study, Zhang et al. formulated microneedle patches with combinations of hair-derived hierarchical microparticles (HMPs) for reactive oxygen species scavenging, well-known growth factors VEGF & bFGF for angiogenesis, and zinc oxide (ZnO) for antibacterial effects [41]. ZnO particles were measured to be 5 nm; the other microneedle patch components’ sizes were not specified [41].

*Exosome Delivery Methods in Spinal Cord Injury Repair and Wound Healing* 

Two studies sought to investigate specially engineered microneedle patches loaded with MSC-derived exosomes and their effects on spinal cord injuries [29,31]. However, Fang et al. noted the difference between particle release from microneedle patches with isolated exosomes against microneedle patches loaded with full MSCs. The study analysis concluded that direct invasion of full MSCs into the spinal cord of rats allowed for sustained release of exosomes, measured at 2-4 x 10^9 particles every two days over 14 days [29]. In comparison, direct invasion of isolated exosome particles demonstrated a sudden drop in particle release, dropping from more than 8x10^9 particles on day 2 to less than 2x10^9 particles by day 14 [29].

Han et al. compared two differences in therapeutic effects between two types of harvested exosomes termed 3D-Exos and 2D-Exos [31]. Retention levels of exosomes delivered through microneedle patches were compared to exosomes injected directly into the spinal tissue. Immunofluorescence confirmed that exosomes delivered via microneedle assay had a larger retention rate compared to exosomes administered by needle injection into the spinal tissue [31]. Furthermore, the therapeutic effects of exosomes were confirmed by increased M1 to M2 macrophage polarization, increased neuroprotection, and prevention of glial scar formation [31].

Studies that assessed wound healing in diabetic rats unanimously concluded that exosome delivery loaded into microneedle patches yielded the best results [30,34,38,41]. Zeng et al. used M2 macrophage-derived exosomes (MEs) incorporated into a double-layer microneedle-based wound dressing system (MEs@PMN). Areas of wound remaining after the treatment period were 17.43% for rats treated with MEs@PMN and 5.01% for rats treated with MEs@PMN + heat [38]. Another study showed microneedle patches, composed of silver and isolated MSC-derived exosomes, healed wounds infected with S. aureus by approximately 100%, while microneedle patches fabricated with only exosomes and without silver healed wounds by approximately 85% [30]. A third study showed that diabetic wounds treated with MSC-exosome-loaded indwelling microneedle patches yielded a wound closure rate of 95.96% ± 3.93% on day 12 following treatment [34].

Zhang et al. conducted a study that demonstrated how microneedle patch nanoparticle combinations can display similar wound healing results to exosomes. Microneedle patches with the ZnO-HMP-VEGF&bFGF composition and the addition of near-infrared irradiation (NIR) caused increased temperature in diabetic rats’ fingertips and marked regeneration of induced full-thickness skin wounds [41]. NIR was applied to mice wounds at a wavelength of 808 nm and area of 0.25 W/cm^2^ every six minutes per time, five times a day [41].

Finally, two of the studies discussed demonstrated an increase in collagen deposition after treatment. Qualitative analysis by Zeng et al. revealed that the percent collagen deposition was highest in the rats treated with MEs@PMN + heat, at 63.83% [38], while study results of Zhang et al. showed that ZnO-HMP-VEGF&bFGF microneedle patches + NIR yielded approximately 70-80% collagen expression [41].

Exosome Delivery Methods in Cosmetic Skin Implications

Four studies assessed exosome delivery for cosmetic purposes [22,28,35,39]. Three of these used microneedling for exosome delivery [28,35,39], while Chernoff et al. used topical exosome application with an injectable calcium hydroxylapatite (CaHA) [27].

Bui et al. utilized microneedle patches coated with hyaluronic acid (HA) to deliver exosomes derived from human adipose stem cells and found that exosome-loaded microneedle patches caused dermal layer thickness to increase from 510 +/- 47 to 715 +/- 35 um [35]. You et al. investigated intradermal delivery of COL1A1 mRNA-encapsulating exosomes using hyaluronic acid-formulated microneedle patches and needle injections; finding similar results with the COL1A1 mRNA exosome microneedle method caused the highest derma thickening and collagen fiber content in the photoaged mice skin compared to control saline injections at one month (180.14 ± 21.46 μm COL1A1-EV MN vs 96.61 ± 14.00 μm Saline) and two months (154.88 ± 8.27 μm COL1A1-EV MN vs 109.25 ± 10.86 μm Saline) [39]. Park et al. explored exosome delivery by topical application with exosomes containing a topical solution (HACS) and microneedling for facial skin aging. The study found that skin elasticity increased by 11.3%, while the control side showed a 3.3% decrease. Skin hydration increased by 6.5% on the HACS side compared to a 4.5% increase on the control side. The melanin index decreased by 9.9% on the HACS side and 1.0% on the control side [28].

Chernoff et al. used topical dermal-infused exosomes with injected CaHA. Of the patients receiving dermal infusion followed by CaHA injections, 16 of 20 reported being very satisfied, and 4 of 20 reported being satisfied [27].

Exosome Utilization for Improved Delivery of Therapeutics

Two studies presented comprehensive experiments in improving the delivery of therapeutics utilizing exosomes [32,37]. Song et al. suggested an improved route of delivery for Ziconotide, a pain medication, that incorporated a microneedle patch that encapsulated Ziconotide within boneole-modified liposomes (LIPS) fused with exosomes [32]. Boneole is an agent used in Chinese medicine to facilitate substance transport across the blood-brain barrier (BBB) [32]. The study noted that the paw withdrawal method, a measure for analgesic effect, improved 50% following the Ziconotide treatment [32]. Additionally, the microneedle patch formulated with exosomes and Ziconotide prolonged the treated rats’ paw-withdrawal threshold for six hours, confirming the successful transport of Ziconotide across the BBB [32].

Yerneni et al. discussed improving skin-targeted delivery through albumin binding, exosome encapsulating, and dissolvable microneedle delivery. The study investigated the anti-inflammatory effects of curcumin and its application in a wide range of skin diseases. The curcumin-albumin-extracellular vesicle/exosome composition (CA-EVs) was tip-loaded into dissolvable microneedle arrays [37]. A psoriasiform inflammatory response was induced.

Comparisons were made between the topical administration of CA-EVs and intradermally injected CA-EVs. “Unloaded” exosomes not loaded with curcumin were also studied. The effectiveness of each of the exosome delivery methods was quantified by measuring the reduction in bacterial lipopolysaccharide inflammatory markers along with Imiquimod-induced skin thickness.

Microneedle CA-EVs reduced LPS inflammatory markers within 3-6 hours following treatment [37]. “Unloaded” exosomes applied with a microneedle patch did not have an anti-inflammatory effect. Topical CA-EVs and “unloaded” exosomes did not have a therapeutic effect in reducing skin thickening caused by Imiquimod [37]. Intradermal injection of CA-EVs reversed inflammation caused by Imiquimod, and CA-EV microneedle assays reduced skin thickness back to healthy levels. Topical CA-EV had minimal impact on thickness. Intradermal CA-EV injection and CA-EV microneedle assay had similar reduced inflammatory marker levels near markers detected in healthy skin [37].

Exosome Delivery Methods in Hair Growth Studies

Four studies involving exosome delivery have examined hair regrowth in rats [33,36,40,42]. Lin et al. used microneedle patches containing nanoparticle doses of rapamycin 0.1 μg and epigallocatechin gallate 4.0 μg to promote hair growth within seven days [40].

Zhao et al. investigated an LPN-loaded dissolving microneedle patch for transdermal miR-218 delivery. The miR-218 expression level in mice treated with microneedle patches reached 1.33 μg/mL in the first few hours, four times higher than the topical gel nanoparticle formulation [42].

Yang et al. focused on a therapeutic microneedle patch with a detachable base delivering human bone marrow MSC-derived exosomes and UK5099. The microneedle patch initiated hair regrowth starting at day 2 of administration, while subcutaneous exosome injections did not initiate hair growth until days 8-10 [33].

Shi et al. utilized a microneedle patch with swellable PVA needle tips and a HA substrate. After 15 days, the microneedle application of CL + EXO (clobetasol and adipose-derived stem cell exosomes) showed a 99% hair regeneration rate, while subcutaneous injections and topical minoxidil had rates of 85% and 77%, respectively [36]. 

A summary of the articles included in this review is reported in Table 1.

**Table 1:** Summary of the Articles Included in the Review (N = 16) EV@MN: Extracellular vesicles loaded in microneedles; LPNs: Lipid-polymer hybrid nanoparticles; MN: Microneedling; MSC: Mesenchymal stem cell; ROS: Reactive oxygen species; DFU: Diabetic foot ulcer; MEs@PMN: M2 macrophage-derived exosomes encapsulated in a polymicroneedle-based wound dressing system; M2 phenotype: Anti-inflammatory macrophage phenotype; EV: Extracellular vesicle; MRNA: Messenger ribonucleic acid; DMNA: Dissolvable microneedle arrays; CA-EV: Curcumin-albumin extracellular vesicles; NF-κβ: Nuclear factor kappa beta; BOR-modified LIPS: Borneol-modified liposomes; ZIC: Zinc; BBB: Blood-brain barrier; CL: Chitosan lactate; ADSC: Adipose-derived stem cells; HACS: Human ASCE-containing solution; DMN: Dissolving microneedles; EGCG: Epigallocatechin gallate; Β-catenin: Beta-Catenin; P-AKT: Phosphorylated AKT; SCI: Spinal cord injury; 3D-Expo: Three-dimensional culture exosomes; MSC-exos: Mesenchymal stem cell-derived exosomes; AgNPs: Silver nanoparticles; MN-MSC: Microneedling with mesenchymal stem cells; MSC-derived EV: Mesenchymal stem cell-derived extracellular vesicles; NO: Nitric oxide; LED: Light-emitting diode; CaHA: Calcium hydroxyapatite

Author	Study Design	Sample	Primary Outcomes
Bui et al. (2022) [35]	Quasi-experimental	Twenty six-week-old mice, divided into five groups, with each group containing four mice	Significant increase in dermal thickness, collagen production, and fibroblast proliferation following EV@MN treatment compared to other delivery methods.
Zhao et al. (2022) [42]	Randomized Control Trial	Six 6–8-week-old mice, divided into two groups, with each group containing three mice	Incorporating LPNs into MN patches provides a targeted and minimally invasive delivery method, which could be relevant for exploring exosome delivery to specific skin layers.
Zhang et al. (2023) [41]	Randomized Control Trial	Twelve eight-week-old male rats, divided into four groups, with each group containing three rats	A novel approach of utilizing light-controlled MN for drug delivery to effectively eliminate ROS, promote angiogenesis, and resist bacterial infection in DFU.
Zhang et al. (2023) [34]	Randomized Control Trial	Twelve diabetic rats, divided into four groups, with each group containing three rats	MSC-exosome-loaded indwelling microneedles significantly promote wound healing in diabetic rats, with faster and more effective wound closure, improved tissue regeneration, enhanced collagen deposition, and reduced inflammation compared to other treatments.
Zeng et al. (2023) [38]	Randomized Control Trial	Four eight-week-old female rats, divided into four groups	MEs@PMN application increased macrophage polarization towards the M2 phenotype in vitro, suppressed inflammation, and promoted vascular regeneration to accelerate diabetic wound healing.
You et al. (2023) [39]	Randomized Control Trial	Twenty-four female mice, divided into six groups, with each group containing four mice	EVs containing mRNA for collagen production can effectively promote collagen formation and reduce wrinkle formation in photoaged mouse skin.
Yerneni et al. (2022) [37]	Quasi-experimental	Twenty 12-week-old male and female rats, divided into five groups, with each group containing four rats	dMNA-delivered curcumin-loaded extracellular vesicles (CA-EVs) effectively enhance drug delivery, inhibit NF-κβ activation, and reduce inflammation in both in vitro and in vivo models of skin inflammation.
Yang et al. (2019) [33]	Quasi-experimental	Twenty-one seven-week-old mice, divided into seven groups, with each group containing three mice	Keratin-based MN patch significantly enhanced hair regrowth by activating hair follicle stem cells, outperforming subcutaneous exosome injections and topical treatments.
Song et al. (2023) [32]	Randomized Control Trial	Five six-week-old male rats and mice, divided into five groups	BOR-modified LIPS fused with exosomes from MSCs and loaded with ZIC improved the ability of crossing the BBB and provide analgesic effects on various pain distributions
Shi et al. (2022) [36]	Randomized Control Trial	Twenty-four seven-week-old male mice, divided into four groups, with each group containing six mice	A drug-free MN patch containing CL and exosomes from ADSCs facilitated hair growth through regulation of the hair follicle cycle; significantly promoted hair regeneration within 7 days compared to topical minoxidil administration; anti-bacterial properties of CL decreased infection risk.
Park et al. (2023) [28]	Randomized Control Trial	Twenty-eight human subjects with facial skin aging underwent a split-face experiment, with one side receiving the treatment and the other serving as the control	MN combined with HACS significantly improved facial skin aging, wrinkles, elasticity, hydration, and pigmentation compared to microneedling with saline, with more pronounced collagen and elastic fiber density in histological evaluations.
Lin et al. (2022) [40]	Quasi-experimental	Forty-five mice, divided into nine groups, with each group containing five mice	DMN co-loaded with rapamycin and EGCG effectively penetrated the skin and showed targeted drug delivery, superior hair regeneration, and hair follicle activation and growth through enhanced β-catenin and p-AKT expression.
Han et al. (2022) [31]	Quasi-experimental	Fifteen mice, divided into five groups, with each containing three mice	Exosomes derived from MSCs cultured in three-dimensional environments (3D-Exo) exhibit superior therapeutic efficiency for SCI repair compared to conventional 2D-derived exosomes. A 3D-exohydrogel MN array patch enabled controlled in situ exosome release, reducing inflammation and glial scarring.
Gan et al. (2022) [30]	Randomized Control Trial	Thirty-two rats, divided into four groups, with each group containing eight rats	A multifunctional MN patch, composed of MSC-exos and AgNPs, significantly accelerated diabetic wound healing by relieving inflammation, promoting angiogenesis, and suppressing bacterial infection.
Fang et al. (2023) [29]	Randomized Control Trial	Fifty-seven rats, divided unevenly into six experimental groups: Control (n = 9), MN (n = 8), Gel-EV (n = 9), Gel-MSC (n = 10), MN-EV (n = 10), and MN-MSC (n = 11)	MN-MSC patch, which facilitated sustained delivery of MSC-derived EVs, effectively reduced inflammation, promoted tissue repair, and protected axons in a spinal cord injury model. Treatment led to improved functional recovery, as seen by better hindlimb motor function, higher BBB scores, and increased axonal survival and regrowth compared to other treatments.
Chernoff et al. (2023) [27]	Case Series	The study enrolled 40 patients (35 females, 5 males, ages 34–72), divided into eight groups, with each group containing five people	Exosome dermal infusion, enhanced with NO, ultrasound, and LED therapy, promoted skin regeneration. Skin priming with an exosome mixture prior to CaHA injections accelerates and enhances results.

Discussion

Main Findings

This scoping review outlines various exosome delivery mechanisms and techniques with the potential to advance the current landscape of alopecia treatments. The 16 studies reviewed included one or more methods of exosome delivery, such as intradermal needle injection, microneedle patch, topical application, and topical application with a secondary assistive device. Critical appraisal of the articles provided valuable insights into the quality of the studies, limitations, and gaps for future research. Additionally, this review offers some insight into the possible sources that exosomes can be derived from. The articles showcased how exosomes can help aid not only hair follicle regeneration but also wound healing, spinal cord injury repair, and collagen regeneration for cosmetic implementations. Finally, a few studies addressed how exosomes can be tailored to deliver desired cargo into target tissues. This discussion section highlights the exosome delivery methods that can be considered for future developments of hair follicle regeneration for non-cicatricial forms of alopecia. 

Diversity of Exosome Delivery Methods and Their Effectiveness

**Figure 2:**
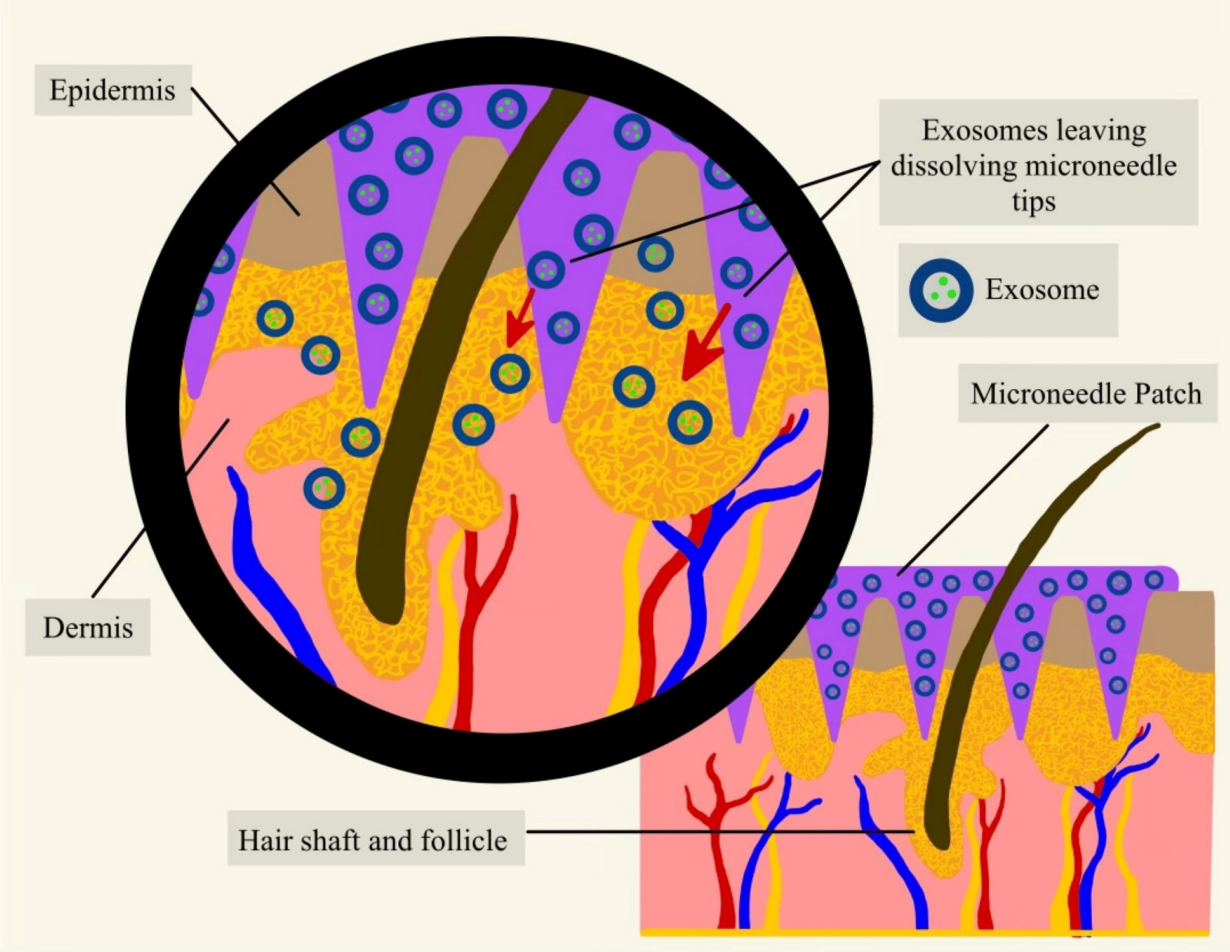
Microneedle Patch Delivering Exosomes into the Skin Image Credit: Sarah Schaffer

Microneedle patches were the most observed device researchers designed to administer exosomes into target tissues. Figure 2 illustrates the mechanism of a microneedle patch dissolving into the skin to release exosomes. The microneedles (purple structures) penetrate the epidermis, allowing the encapsulated exosomes (blue circular structures) to diffuse into the underlying tissue. The exosomes travel toward the sebaceous glands and surrounding cells (indicated by red arrows), facilitating targeted delivery for therapeutic or regenerative effects.

Frequently, microneedle patches were studied in comparison to other exosome delivery methods, such as intradermal injections and topical application. For example, Lin et al. designed a dissolvable microneedle patch with nanoparticles loaded with rapamycin and epigallocatechin gallate and compared the microneedle patches with the topical application of the nanoparticles [40]. A second study explored the effects of a lipid-polymer hybrid nanoparticle-loaded microneedle patch’s ability to promote hair regrowth by transdermal miR-218 delivery [42]. Both studies concluded that therapeutic agents loaded into nanoparticles and administered into the dermis of mice with a microneedle patch produced better hair growth when compared to topical formulations [40,42]. Specifically in the work of Lin et al., microneedle patches with rapamycin doses of 0.1 dose ug and epigallocatechin gallate doses of 4.0 ug produced observable hair growth on day 7 compared to the topical applications of rapamycin and epigallocatechin gallate nanoparticle compositions [40]. In the research of Zhao et al., hair regrowth following the shaving of mice’s backs was first observed on day five, along with significant hair regrowth by day 13 in comparison to the cohorts of mice treated with topical lipid-polymer hybrid nanoparticle formulations [42].

The efficacy of findings of Zhao et al. demonstrated how miR-218 expression levels in mice treated with microneedle patches were four times higher than miR-218 expression levels in mice treated with topical gel nanoparticle formulations [42]. This finding suggests the potential for improving current topical alopecia treatments by formulating them in a manner that facilitates dermal penetration using a microneedle patch.

The unique characteristics of microneedle patch design of Lin et al. are that doses of rapamycin and epigallocatechin gallate were smaller than the drug doses administered to mice skin in the topical form. For example, in the rapamycin microneedle patch groups, doses tested were 0.001 μg, 0.01 μg, 0.1 μg, 1 μg, and 10 μg [40]. Rapamycin lecithin topical gel doses were 0.2 μM, 2 μM, and 20 μM [40]. This shows that microneedle patches can assist in low-dose drug delivery without compromising the effectiveness of treatment that may be seen at higher doses. It is important to note that the two previously mentioned studies and a third study included in our scoping review did not use exosomes as a mode of therapeutic delivery [40-42]. Zhang et al. administered ZnO to diabetic rat wounds with microneedle patches [41]. Nanoparticles and inorganic compounds like ZnO possess exosome-like properties. First, nanoparticles and ZnO are similar in size to the size range of exosomes (~40 to 160 nm) and thus can penetrate target tissues to the degree that exosomes do [16,40-42]. Additionally, nanoparticles can slow the degradation and oxidation of loaded contents similarly to exosomes [16,40,42]. These similarities help bridge the findings in these studies to the advancement of alopecia treatments using exosomes.

Studies that utilized exosomes, like Yang et al. and Shi et al., also demonstrated superior exosome delivery via microneedle patch for hair regrowth and generation. However, both studies compared the microneedle patch administration of exosomes to the subcutaneous injection of exosomes. Shi et al. took one step further and compared the microneedle patches and injectable exosomes to topical minoxidil and concluded that exosomes loaded with clobetasol delivered with a microneedle patch had almost complete hair regeneration by day 15 compared to the exosome injections and topical minoxidil [36]. For Yang et al., the results of the UK5099 topical application were studied, too. Still, the microneedle patch with exosomes + UK5099 caused observed hair regrowth within six days [33]. The effects of the microneedle patch-initiated hair regrowth starting at day 2, and subcutaneous exosome injections showed hair growth by days 8-10 [33]. Both studies help emphasize how exosome delivery via microneedle patch is effective, and the use of exosomes may outperform current alopecia treatments.

Exosome Sources and Applications

Another theme this scoping review outlines is the diversity in sources that exosomes were isolated from. Furthermore, exosomes were selected by researchers depending on the study’s design application of exosomes. Applications include hair regrowth, wound healing, spinal cord injury repair, and cosmetic purposes.

MSCs were the most frequently used source for exosome isolation [27,29-34]. Some studies used MSCs as a source because MSC-exosomes were cited to have regenerative and anti-inflammatory properties desirable for their specific study designs [27,30,31]. Other cited justifications for the selection of MSCs included their long-term survival and sustained release of exosomes [29], the activation capability of fibroblasts, vascular endothelial cells, and macrophages by MSC-derived exosomes [34], and the biocompatibility of MSCs [32]. Human adipose-derived stem cells were also used in some studies [27,28,35]. Two sources cited that adipose-derived stem cells produced exosomes with regenerative renewal capacities that have been shown to have promising therapeutics for a variety of diseases [28,35]. It is important to note that these two studies also focused on exosome applications in skin aging [28,35]. However, in the study on hair regrowth therapy by Shi et al., adipose-derived stem cells were utilized without a clear specification of why exosomes from adipose-derived stem cells were chosen over exosomes from other sources [36]. This connection between these three studies introduces yet another possible exosome isolation source for alopecia treatment.

Finally, a few studies used less common sources of exosomes, such as macrophages [37,38] and human dermal fibroblasts [39]. One study examining the anti-inflammatory benefits of curcumin did not specify why macrophages were desired exosome sources, but researchers did dictate that exosomes were desired for the researchers’ study design because exosomes were able to omit the poor stability and bioavailability of free curcumin [37]. Zeng et al. specified that macrophage-derived exosomes produced anti-inflammatory capabilities that were desired for their study design relating to wound healing in diabetic rats [38]. Finally, You et al. utilized exosomes derived from human dermal fibroblasts to encapsulate the COL1A1 mRNA for collagen replacement therapy in photo-aged rats [39]. These unique examples of exosome isolation sources further emphasize the diversity of options that may be available for incorporation into future alopecia treatments.

Another topic of interest highlighted in all the studies was the application in which exosomes were used and how these study designs can relate to the development of alopecia treatments. Four of the studies evaluated hair regrowth in response to treatments of exosomes, and all four studies demonstrated hair regrowth with microneedle patches within at most seven days [33,36,40,41]. The therapy formulations and doses in these studies warrant further investigation as to which formulation can optimize treatments for alopecia patients. The findings in these studies demonstrate how the use of exosomes for hair regrowth opens possibilities for breakthrough treatments not yet available to the patient population.

The effects of exosome-formulated therapies on wound healing were another common topic researchers studied [30,34,38,41]. Studies demonstrated that microneedles combined with therapeutic agents loaded into exosomes could result in healing patterns beginning in a day [38], increased collagen expression within healed tissue [41], and a faster near 100% closure rate compared to other treatments [30,34]. Understanding the mechanisms behind exosomes’ efficacy in wound healing may be of importance when developing hair regenerative treatments. Similarly to wound healing, some studies observed exosome effects on spinal cord injury repair [29,31]. The studies observed that exosomes delivered using microneedle patches were able to cause decreased glial scar tissue formation, promote angiogenesis, and improve axon survival [29,31]. These findings highlight possible neurological aspects that may be relevant to alopecia development. However, this scoping review did not seek to find the neurological relationships between hair regrowth for alopecia.

Finally, four studies looked at exosome use in skin aging [27,28,35,39]. Dermal thickness and collagen expression in mice were increased in microneedle applications of exosomes [35,39], and the topical application of a therapeutic solution with exosomes improved skin elasticity and skin hydration in humans when administered into the skin with a handheld microneedle device [28]. Chernoff et al. utilized a dermal infusion technique to enhance topical exosome penetration into the skin [27]. These studies did not examine hair regrowth, but since dermal tissue was the medium exosomes were delivered into, the findings in all four studies can be considered when formulating alopecia treatments that can be administered into the scalp dermis.

Future Directions and Research Gaps

This review examined various exosome delivery methods and their therapeutic potential. However, the majority were animal studies with experiments conducted on mice and rats. Of the two studies that involved human subjects, hair regrowth was not an isolated variable. Park et al. investigated the effects of using topical adipose tissue cell-derived exosomes (ASCEs) in conjunction with a handheld microneedling device on 28 human participants but looked (only) at the therapeutic effects on facial skin aging [28]. Although this was one of the first human clinical studies to use ASCEs for facial skin aging, the number of participants was small, and the follow-up period (six weeks) was relatively short [28]. Alongside, Chernoff et al. also investigated exosome therapeutics for enhanced tissue biostimulation using 40 human participants [27]. Exosomes were derived from MSCs and topically applied in conjunction with CaHA. Though multiple skin markers were assessed, such as wrinkles, pores, and oiliness, the effects on hair regrowth were not analyzed [27].

The subjects in all the studies imitated alopecia by shaving the hair of rat or mouse subjects, creating an “induced” hair loss [33,36,40,42]. Although exosomes can still be delivered, these models do not accurately depict the non-cicatricial forms of alopecia. Future research should focus on testing exosome delivery in clinically relevant alopecia models rather than artificially induced hair loss to accurately test the effects of the exosomes delivered.

Additionally, multiple exosome sources were explored, such as MSCs, human-dermal fibroblast-derived macrophages, macrophage-derived exosomes, etc. Further research studies are needed to explore which source of exosomes is the most effective in treating non-cicatricial forms of alopecia by comparing these exosome source forms against each other.

Current hair regrowth treatments for alopecia patients include minoxidil, finasteride, and biotin, yet few studies that investigated the role of exosome therapeutics utilized these current hair treatments in the derivation of their exosome sources. Further research is thus needed to enhance our understanding of selecting the best therapeutic source when isolating exosomes.

Limitations of the Articles Reviewed

The majority of studies were animal-based, with only two human studies that did not directly focus on hair regrowth. The animal models used induced hair loss by shaving, which does not accurately represent the complex pathophysiology of non-cicatricial alopecia in humans. None of the studies comprehensively compared different exosome sources to determine the most effective for treating alopecia. Most studies utilized microneedle patch delivery methods, potentially limiting the generalizability of findings to other delivery techniques. Additionally, the studies lacked standardization in exosome isolation, purification, and characterization, which poses a significant challenge in interpreting and comparing results across different research efforts.

Limitations of the Scoping Review

The scoping review itself had several inherent limitations. The search was restricted to English-language publications between 2018 and 2023, potentially missing relevant studies in other languages or published outside this timeframe. The review's methodology relied on a limited number of databases, which might have excluded some relevant research. The inclusion of only 16 studies after an initial search of 1,087 citations suggests a narrow focus and potentially missed important research. Additionally, the search terms used to gather studies may have left out publications using different terminology to describe exosomes. Furthermore, the review did not conduct a meta-analysis or perform a detailed comparative analysis of the included studies, which could have provided more nuanced insights into the potential of exosome-based treatments.

## Conclusions

This review emphasized the extent to which various exosome delivery mechanisms can be utilized to work synergistically with existing treatments for alopecia in humans. Exosomes have been suggested to be an effective component in therapy development for not only hair follicle regeneration but also for wound healing, spinal cord injury repair, and cosmetic skin applications such as the improvement of skin aging and firmness. This review identified possible exosome-related advancements for non-cicatricial alopecia and the various mechanisms by which exosomes were delivered are relevant. Additionally, the reviewed studies demonstrated the diversity of possible exosome isolation sources, such as MSCs, human adipose-derived stem cells, macrophages, and human dermal fibroblasts. Several exosome delivery methods were documented among studies, including the use of microneedle patches, topical formulations, intradermal injections, and secondary assistive devices. Microneedle patches containing therapeutic agent-loaded exosomes were a common delivery method that reigned successful over other delivery methods. While further research is warranted, microneedle patches laced with exosomes may be an ideal vessel to assist with hair regeneration in non-cicatricial forms of alopecia in humans. The importance and possible benefits of such an advancement could help lead to faster results for patients with fewer side effects.

## Appendices

**Table 2:** Key Search Terms (1,087 results)

Boolean Operator	Search Term
	(minoxidil':ab,ti,kw OR 'finasteride':ab,ti,kw OR 'duasteride':ab,ti,kw OR 'spironolactone':ab,ti,kw OR 'cyproterone acetate':ab,ti,kw OR 'corticosteroids':ab,ti,kw OR 'janus kinase inhibitor':ab,ti,kw)
OR	(('exosome':ab,ti,kw OR 'exosome therapy':ab,ti,kw OR 'extracellular vesicle':ab,ti,kw)
OR	('alopecia':ab,ti,kw OR 'non-cicatricial alopecia':ab,ti,kw OR 'hair loss':ab,ti,kw OR 'hairloss':ab,ti,kw OR 'hair regeneration':ab,ti,kw OR 'non scarring alopecia':ab,ti,kw OR 'hair regrowth':ab,ti,kw OR 'hair growth':ab,ti,kw)))
AND	(('microneedle':ab,ti,kw OR 'micro needling':ab,ti,kw OR 'low level laser therapy':ab,ti,kw OR 'laser assisted thus drug deliver':ab,ti,kw OR 'platelet rich plasma':ab,ti,kw OR 'platelet rich fibrin':ab,ti,kw OR 'autologous conditioned plasma':ab,ti,kw)
AND	(('exosome':ab,ti,kw OR 'exosome therapy':ab,ti,kw OR 'extracellular vesicle':ab,ti,kw) OR ('alopecia':ab,ti,kw OR 'non-cicatricial alopecia':ab,ti,kw OR 'hair loss':ab,ti,kw OR 'hairloss':ab,ti,kw OR 'hair regeneration':ab,ti,kw OR 'non scarring alopecia':ab,ti,kw OR 'hair regrowth':ab,ti,kw OR 'hair growth':ab,ti,kw).
